# Chitosan–Graphene Oxide Composite Membranes for Solid-Phase Extraction of Pesticides

**DOI:** 10.3390/ijms22168374

**Published:** 2021-08-04

**Authors:** Ilaria Silvestro, Clarissa Ciarlantini, Iolanda Francolini, Pierpaolo Tomai, Alessandra Gentili, Chiara Dal Bosco, Antonella Piozzi

**Affiliations:** Department of Chemistry, Sapienza University of Rome, 00185 Rome, Italy; ilaria.silvestro@uniroma1.it (I.S.); clarissa.ciarlantini@uniroma1.it (C.C.); iolanda.francolini@uniroma1.it (I.F.); pierpaolo.tomai@uniroma1.it (P.T.); alessandra.gentili@uniroma1.it (A.G.); chiara.dalbosco@uniroma1.it (C.D.B.)

**Keywords:** chitosan–graphene oxide membranes, solid-phase extraction, pollutants, chitosan, graphene oxide

## Abstract

Solid-phase extraction (SPE) coupled to LC/MS/MS analysis is a valid approach for the determination of organic micropollutants (OMPs) in liquid samples. To remove the greatest number of OMPs from environmental matrices, the development of innovative sorbent materials is crucial. Recently, much attention has been paid to inorganic nanosystems such as graphite-derived materials. Graphene oxide has been employed in water-purification processes, including the removal of several micropollutants such as dyes, flame retardants, or pharmaceutical products. Polysaccharides have also been widely used as convenient media for the dispersion of sorbent materials, thanks to their unique properties such as biodegradability, biocompatibility, nontoxicity, and low cost. In this work, chitosan–graphene oxide (CS_GO) composite membranes containing different amounts of GO were prepared and used as sorbents for the SPE of pesticides. To improve their dimensional stability in aqueous medium, the CS_GO membranes were surface crosslinked with glutaraldehyde. The composite systems were characterized by Fourier transform infrared spectroscopy, scanning electron microscopy, thermogravimetric analysis, swelling degree, contact angle, and mechanical measurements. As the GO content increased, a decrease in surface homogeneity, an improvement of mechanical properties, and a reduction of thermal stability of the CS-based membranes were observed. The increased dimensional stability in water, together with the presence of high GO amounts, made the prepared composite membranes more efficacious than the ones based just on CS in isolating and preconcentrating different hydrophilic/hydrophobic pollutants.

## 1. Introduction

Pesticides are among the most consequential environmental pollutants responsible for the contamination of soil, atmosphere, and aquatic bodies as a result of human activities. In the last decades, the enhanced demands for food production and health-protection programs have led to an uncontrolled use of pesticides, with negative consequences for the environment and human health [1]. The term pesticide usually refers to several types of substances scheduled according to their chemical structure, toxicity, and activity. This wide class of chemical agents mainly includes insecticides, fungicides, and herbicides. Features such as remarkable toxicity and long persistence in the environment (bioaccumulation) make pesticides extremely dangerous. Moreover, the environmental impact of such substances can increase, as pesticides might easily migrate in aquifers and enter the food chain, resulting in an accumulation in organisms [2,3]. Pesticides released into the environment can also be transformed, leading to the formation of metabolites that often are more toxic than the original substances [4]. In fact, a correlation was found between the use of pesticides and the increase of health conditions such as allergies, cancer, malformation, and DNA mutation [5,6]. Pesticides, as well as drugs, plasticizers, and flame-retardants, belong to the class of organic micropollutants (OMPs), which are usually found in the environment at trace levels. Since their environmental persistence could compromise agricultural and human consumption of water, the determination and quantification of OMPs in aquatic bodies (groundwater and surface water) have gained widespread attention. In addition, pesticides are present in water bodies at low concentrations; therefore, a specific approach for their determination is necessary.

To this aim, solid-phase extraction (SPE) has been widely used with good results. SPE is a simple and rapid technique, exploiting as the extracting phase a solid material with good affinity for different substances that are usually present into environmental matrices. After their extraction, OMPs can be concentrated by desorption with little amount of mobile phase, reducing the use of organic solvents. Sample preparation is a crucial step for an accurate determination of analytes. To date, great efforts have been made to develop several sorbent materials for SPE using organic, inorganic, and composite materials in order to determine pesticides in environmental samples or foodstuffs. For instance, Özer et al. synthetized poly(divinylbenzene-*N*-methacryloyl-l-tryptophan methyl ester microbeads (PDMAT) for the preconcentration of organophosphorus pesticides (OPPs) from water samples [7], while magnetic covalent organic framework (COF) was employed as magnetic sorbent for OPPs in milk samples [8]. Recently, much attention has been paid to inorganic nanosystems such as graphite-derived materials [9]. Graphene oxide (GO), a 2D material with a honeycomb structure, derives from oxidation and exfoliation of graphite. The excellent mechanical, electronic, and optical properties shown by GO make this material applicable in optic, electronic, and biomedicine fields [10]. Features such as the presence of different functional groups (carboxyl, epoxy, and hydroxyl groups) and large surface area play an important role in its use as a sorbent material. Indeed, it has already been employed in water-purification processes, including the removal of several micropollutants such as dyes, flame-retardants, or pharmaceutical products [11]. To reduce aggregation between graphene sheets, GO can be combined with polymeric systems [12,13,14]. Polysaccharides have gained considerable attention in the preparation of sorbent materials thanks to their unique properties such as biodegradability, biocompatibility, nontoxicity, and low cost. Chitosan (CS) is a biopolymer widely used in different applications such as tissue engineering and agricultural and wastewater treatment [15,16]. CS is obtained by deacetylation of chitin and composed of β-(1→4)-D-glucosamine and *N*-acetyl-D-glucosamine units [17]. The poor resistance of CS in acid media is due to the high hydrophilicity of amine groups. The formation of polymeric networks is a promising way to prevent its dissolution and to enhance its mechanical properties. In this case, the amine groups of CS are suitable reactive sites for the formation of such networks. In fact, physical interactions promoted by anionic crosslinkers (sodium tripolyphosphate, sodium citrate, sulfosuccinic acid, oxalic acid), or covalent bonds obtained by using chemical crosslinkers (genipin, epichlorohydrin, glutaraldehyde), have allowed the broadening of CS applicability [18]. To this aim, nanometric fillers have also been used. For instance, hydroxyapatite can modulate the physicochemical properties (swelling and mechanical performance) of CS, also providing polymers with an improved bioactivity [19,20], while a better antimicrobial activity can be obtained by using silver nanoparticles [21]. Chitosan–graphene oxide composites have been successfully used as proficient sorbent materials for the removal of different pollutants. The results obtained in this field by employing CS_GO systems can be found in a recent review by da Silva Alves et al. [22]. For instance, chitosan/polyacrylamide/graphene oxide nanocomposites (CAGs) containing 20% GO were able to reach the maximum absorption capacity of methylene blue, while a removal efficiency of rifampicin of more than 95% was obtained by applying magnetic CS_GO nanocomposites [23,24].

The development of versatile sorbent materials able to interact with different types of substances is of great importance, since a wide variety of pollutants usually contaminate water compartments. However, an excessively hydrophilic material, such as chitosan, could compromise interactions with nonpolar or hydrophobic pesticides [25].

Therefore, the main goal of this study was to assess the role of GO in developing a versatile sorbent composite system able to adsorb pollutants of different natures. To this aim, to modulate hydrophilicity of the polymer and its affinity towards hydrophobic pollutants, CS was mixed with different amounts of GO (from 1% to 20% *w*/*w*), and composite membranes were obtained by solution casting. To improve their dimensional stability in aqueous medium, the composite membranes were submitted to surface crosslinking with glutaraldehyde. Then, the CS_GO membranes were characterized by infrared spectroscopy, thermogravimetric and mechanical analysis, and swelling and contact-angle measurements. To assess their adsorbent ability, the CS_GO composites were used in preliminary experiments aimed at extracting 20 pesticides of different classes (i.e., herbicides, fungicides, etc.), with different log K_ow_. To the best of our knowledge, such membrane-based systems have never been studied for the simultaneous determination of pesticides.

## 2. Results and Discussion

**Figure 1:**
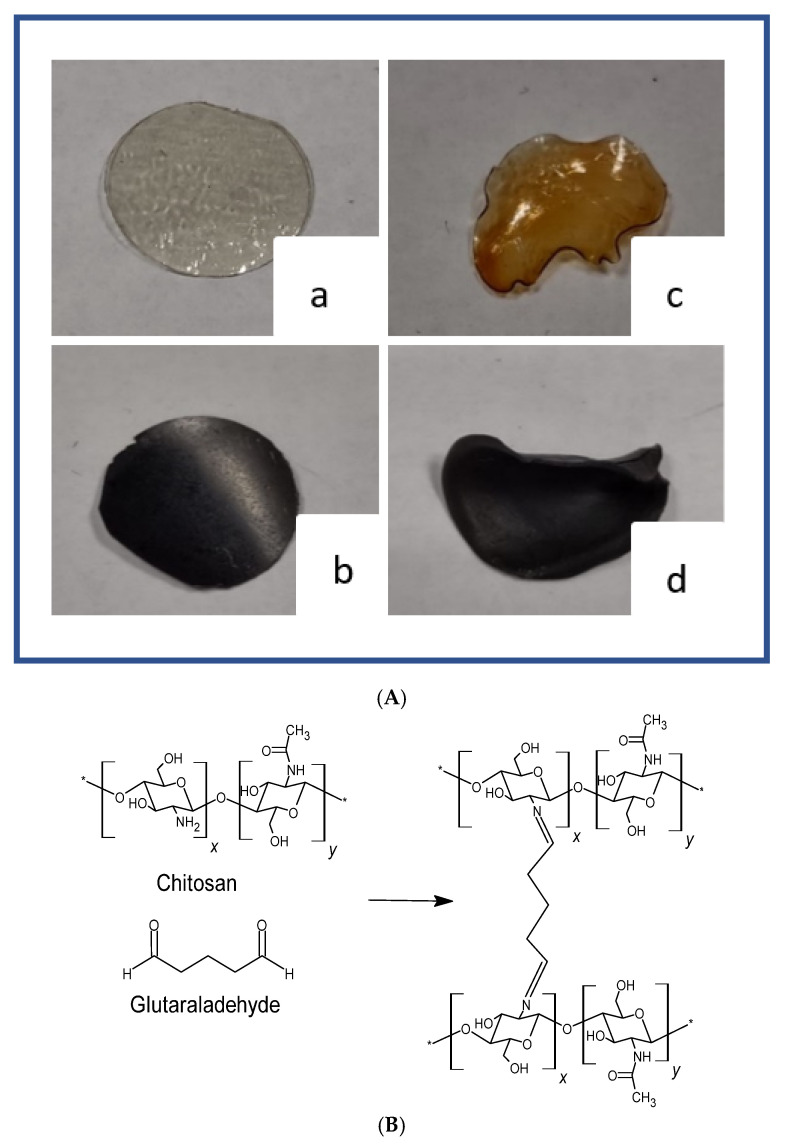
(**A**) Illustration of non-crosslinked CS (**a**) and CS_GO10 (**b**); and crosslinked CS (**c**) and CS_GO10 (**d**). (**B**) Scheme of the crosslinking reaction between chitosan and glutaraldehyde.

SPE is an analytical technique widely used for the determination of trace pollutants. Currently, the development of proficient sorbent materials for SPE is still an open challenge. GO is a nanomaterial that could guarantee good affinity for a wide range of pollutants [26,27,28]. However, complicated recovery operations related to its nanosize restrict GO’s use. To expand the applications of GO, its combination with a polymer matrix such as chitosan could lead to convenient composite materials able to interact with a wider range of pollutants [29,30,31]. Herein, CS and CS_GO membranes were prepared with the solvent casting method, and the influence of GO content on their physicochemical properties was studied. The acronyms for the obtained membranes, along with their physicochemical properties, are reported in Table 1. Since the presence of GO may not be sufficient to stabilize the polysaccharide in an aqueous environment for a very hydrophilic matrix such as chitosan, a crosslinking reaction with glutaraldehyde on preformed membranes was performed. The images of pristine CS and CS_GO composite membranes before and after the crosslinking phase are reported in Figure 1A, while in Figure 1B, a scheme of the crosslinking reaction is depicted. It was noted that the presence of GO strongly influenced the membrane colour, which changed from colourless to intense black.

### 2.1. Scanning Electron Microscopy

**Figure 2:**
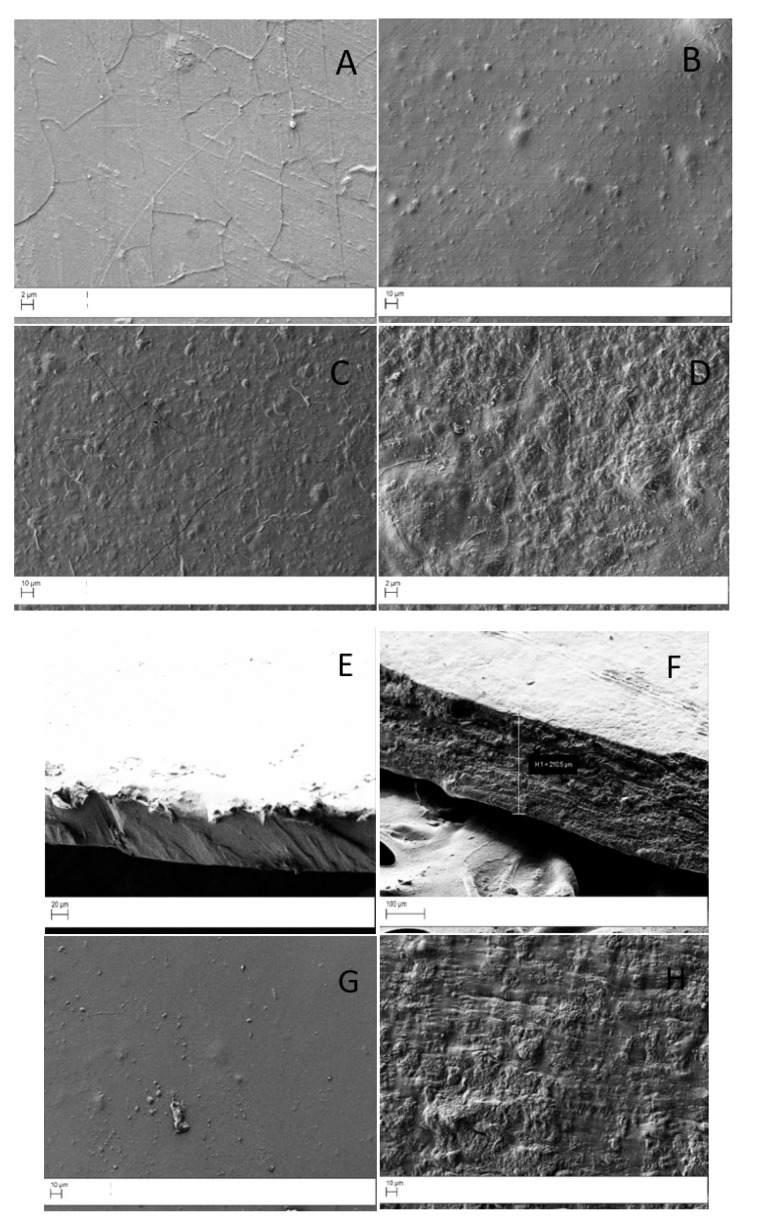
Micrographs of pristine CS (**A**) and CS_GO composites: CS_GO1 (**B**), CS_GO5 (**C**), and CS_GO20 (**D**). Cross-section of pristine CS (**E**) and CS_GO20 (**F**). Micrographs of CS_GLU (**G**) and CS_GO20_GLU (**H**).

To investigate the surface modification of CS film after introduction of GO and to achieve information about filler dispersion into the composite matrices, the surface morphology of the membranes was observed by scanning electron microscopy (SEM). In Figure 2, some representative micrographs of the prepared samples are reported. The surface of pristine CS appeared uniform and smooth, with few wrinkles and grooves (Figure 2A), which increased with the GO content (Figure 2B–D). Indeed, a good miscibility between the two components was visible for the CS_GO1 sample (GO was homogeneously dispersed), while a remarkable filler aggregation was observed for the CS_GO20 sample (Figure 2D). These results were in agreement with those reported in the literature [32,33,34]. For instance, Qian, Xiaowei et al. found segregation phenomena in chitosan–graphene oxide membranes when GO loading was 2% [35]. The presence of GO also affected the bulk membrane morphology. Indeed, the cross-sectional surface of CS was rather smooth (Figure 2E), while the cross-section of the CS_GO20 sample had a layered structure (Figure 2F), with GO sheets enwrapped by CS.

No significant change in the surface morphology was evidenced after the crosslinking with glutaraldehyde for pristine CS and CS composites (Figure 2G,H).

### 2.2. Fourier Transform Infrared Spectroscopy

**Figure 3:**
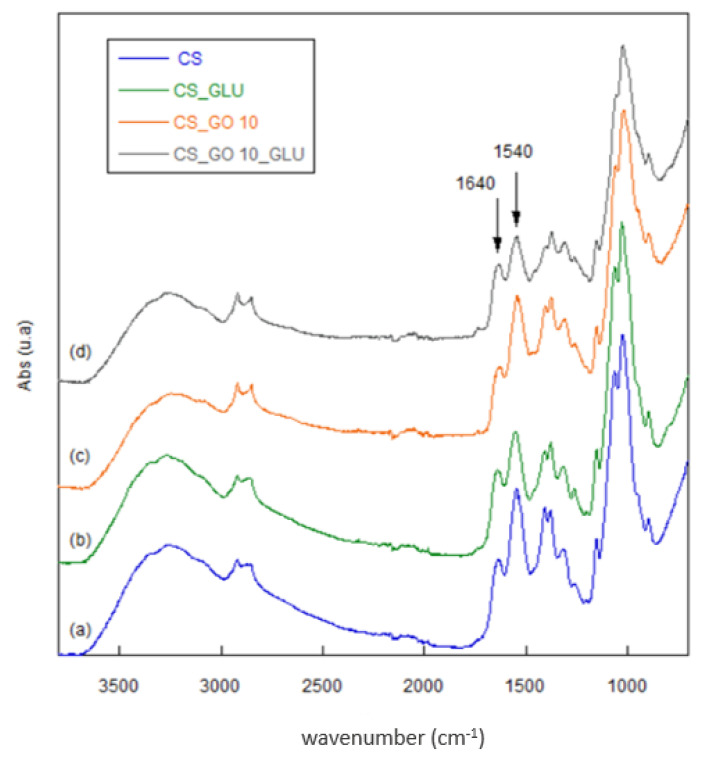
FTIR spectra of pristine CS (a), CS_GLU (b), CS_GO10 (c), and CS_GO10_GLU (d).

To verify the interaction between CS and GO and to confirm the crosslinking reaction with glutaraldehyde, an FT-IR analysis of CS_GO composite materials was carried out. In Figure 3, as an example, the spectra of CS_GO10 and CS_GO10_GLU are compared with the spectra of pristine and crosslinked CS.

The prominent band in the CS spectrum in the range of 3600–3000 cm^−1^ was due to the –OH and NH_2_ stretching, while absorptions around 2920–2852 cm^−1^ were related to the C–H stretching. At 1640 cm^−1^, the C=O stretching of acetylate groups (amide I) is clearly visible, as well as the N–H bending and the C–N stretching of the amide II band at 1550 cm^−1^. Absorption at 1381 cm^−1^ was attributed to symmetric CH_3_ deformation and C-H bending, while the amide III band and CH_2_ wagging absorbed at 1310 cm^−1^. In the range of 1150–1000 cm^−1^ the absorptions due to C–O–C and C–O–H stretching are present, while at 896 cm^−1^, the absorption related to the pyranose ring stretching can be seen [36]. In accordance with previous studies, the spectra of CS_GO composites showed no noticeable differences from the spectrum of pristine CS, nor new peaks [32,37]. However, a slight shift to lower wavenumbers of the C=O peak was observed for the CS_GO composites with respect to CS, because of the hydrogen bond formation and electrostatic interaction between the two components. These findings suggested a good compatibility between the polymeric matrix and the filler. The most common reaction between glutaraldehyde and chitosan is the formation of a Schiff base. The C=N stretching vibration should be detected in the range of 1620–1660 cm^−1^. In our case, the crosslinked and non-crosslinked membranes did not show pronounced differences, since the reaction involved the use of a small amount of crosslinker (0.1% *v*/*v*). Nevertheless, as reported in the literature by Rodríguez-Velázquez et al., the area increase of the band at 1640 cm^−1^ could be related to the formation of the Schiff base [38]. To find evidence of this, the ratio between the area of peaks at 1640 cm^−1^ and 1550 cm^−1^ (amide II) for pristine CS and CS_GO composites, before and after the crosslinking reaction, was determined. As shown in Table 1, after the crosslinking reaction, a slight increase of the A_1640_/A_1540_ ratio was noted for the CS_GOX_GLU composite materials, confirming the surface modification. Moreover, it was observed that such A_1640_/A_1540_ ratio slightly decreased with the increasing GO content, evidencing a lower degree of crosslinking. This was more noticeable for the CS_GO20_GLU sample. In this latter case, a greater number of electrostatic interactions between the GO COOH groups and the CS amino groups most likely occurred, thus reducing the availability of NH_2_ groups for the crosslinking reaction. In addition, the morphological deformation undergone by the samples after reaction with glutaraldehyde was more evidence that the crosslinking reaction had effectively occurred (see Figure 1A,C,D). Finally, the absence of a peak at 1740–1720 cm^−1^, related to C=O carbonylic groups, confirmed the absence of unreacted aldehyde.

### 2.3. Thermogravimetric Analysis

**Figure 4:**
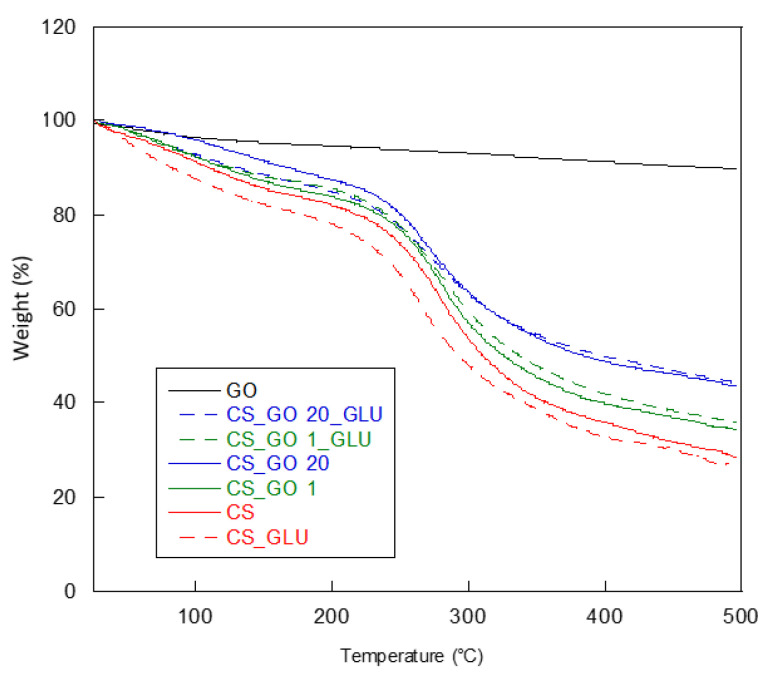
Thermogravimetric curves of pristine CS, GO, and CS_GOX composites before and after the crosslinking reaction.

To determine the thermal stability of CS-based membranes, either containing GO and the crosslinking agent or not, thermogravimetric analysis was carried out. It is known that the nanofiller structure and its dispersion degree in the matrix, as well as chemical interactions with a crosslinker, can affect the polymer thermal stability. TGA curves of the CS, CS_GO1, and CS_GO20 samples, pristine and crosslinked, are reported as an example in Figure 4, while T_d_ (degradation temperature) and weight-loss values are listed in Table 1. All of the samples showed two main stages of degradation. The first stage, in the 25–150 °C range, was due to the evaporation of water that was physically adsorbed and strongly bonded to the polymer, whereas the second stage of degradation, at higher temperatures, was attributed to the complex pyrolysis process of the polysaccharide. Indeed, it was reported that such a process begins with a random split of glycosidic bonds, followed by further decomposition, forming acetic and butyric acids and a series of lower fatty acids (C2, C3, and C6) [39]. As far as the non-crosslinked samples are concerned, an increase of the T_d_ value was observed for CS_GO1 compared to the pristine CS. The reproducibility of the data was verified by performing more than one measure per sample, and there was little variance in the degradation temperatures, which were reproducible below 1 °C for all the samples. This increase confirmed that when a small amount of GO was used, a homogenous distribution of the filler in the polymer matrix could be obtained. The good miscibility of the two components favoured electrostatic interactions and hydrogen bonds between CS and GO, which was responsible for the increased thermal stability of the CS_GO1 sample.

In contrast, the CS_GO5 and CS_GO10 samples showed a T_d_ value approximately equal to that of CS, while the CS_GO20 membrane was found to be less thermally stable. It was most likely that with an increasing GO amount, a segregation of filler occurred, thus disturbing polymer chain packing, with a consequent diminution of the T_d_ value. In the literature, no variation in the CS T_d_ value with the increase of GO content has been evidenced [34,40]. In any case, in most investigations, low percentages of GO were employed, from 0.1 to 6% (*w*/*w*) [34,35,40,41], whereas when higher GO amounts were used (until to 25% *w*/*w*), no thermogravimetric analysis was provided [42]. As far as the degradation temperature of the CS_GOX_GLU composites is concerned, to verify that the surface crosslinking reaction was homogenous, the analysis was performed on several pieces of the same membrane. A diminution of T_d_ was observed with the increase in GO amount. However, T_d_ values were higher than those obtained for the CS_GOX samples. In this case, the high thermal stability of the cross-linked composites was due to the formation of chemical bonds consequent to the crosslinking process. A singular behaviour was shown by the CS_GLU sample that had a T_d_ value lower than the pristine CS. This result was in accordance with previous works in which 0.01% glutaraldehyde was used for the CS crosslinking reaction [39]. The authors reported that when GLU concentration was low, the crosslinking process was unfavourable to hydrogen bond formation, causing a diminution of the T_d_ value. In our work, however, crosslinker concentration was 0.1%, a value higher than that used in the cited study. This behaviour was not observed for the samples containing the filler, since the presence of GO reduced the destabilizing action made by glutaraldehyde. In Table 1, the weight loss for the main two stages of degradation is shown. As previously reported, the first stage of degradation, in the 25–170 °C range, was due to the structural water loss. By analyzing the data in Table 1, the water content of the non-crosslinked samples was found to be influenced by the presence of GO. In fact, CS retained about 16% of its water, while a slight reduction was detected after GO introduction. The lowest water content was found for CS_GO20. The same trend was observed for the CS_GO X _GLU crosslinked composites, in which the crosslinking reaction caused a further reduction in water content. In this case, a singular behaviour also was shown by the CS_ GLU sample, which retained a water amount higher than pristine CS. Neto et al. attributed the increase in water content to a decrease in the ordered structure of CS due to chemical modification caused by the crosslinking reaction [39]. By observing the data measured in the 170–500 °C range, it was possible to note how the presence of GO and the crosslinking reaction reduced the total weight loss of all samples if compared with CS and CS_GLU.

### 2.4. Mechanical Characterization

Sorbent materials for SPE applications must have good mechanical performance, since they must resist several stresses during their use. Graphene oxide is usually employed as a reinforcing filler with the aim to improve the mechanical behaviour of CS [42,43]. Therefore, the effect of GO introduction into the CS membrane, as well as the influence of the crosslinking reaction on the composite materials, were examined. The results are reported in Table 1. Generally, the introduction of GO contributed to the improvement of the mechanical properties of CS, as evidenced by the rise in the elastic modulus values [44]. Specifically, when a low amount of GO was added to the polymer (CS_GO 1 and CS_GO 5), no significant variation was observed with respect to pristine CS. In fact, even if the dispersion was better at low filler content, the GO amount was probably to small to modify the mechanical performance of the CS. In contrast, with an increasing GO quantity, an elastic modulus twice as high as that of pristine CS was obtained (see the CS_GO20 sample). This led to an increase in tensile strength, with a reduction in the elongation at break value of about 45%. As for the influence of the GLU reaction on the mechanical behaviour of the composite film, the crosslinking process negatively affected the mechanical resistance of the composite membranes. Generally, all of the crosslinked samples showed values for the elastic modulus, tensile strength, and elongation at break lower than the CS_GOX samples, suggesting an increase in their brittleness. Particularly, a high fragility was observed for the CS_GLU membrane, enough that it was not possible to perform the mechanical tests. Interestingly, the presence of GO reduced the glutaraldehyde effect. Indeed, the CS_GO_GLU composite membranes were characterized by less fragility (see data in Table 1). These findings were in agreement with the data obtained by IR analysis, in which a lower crosslinking degree with the GO amount increasing was obtained.

### 2.5. Swelling Capacity and Water Contact-Angle Measurements

**Figure 5:**
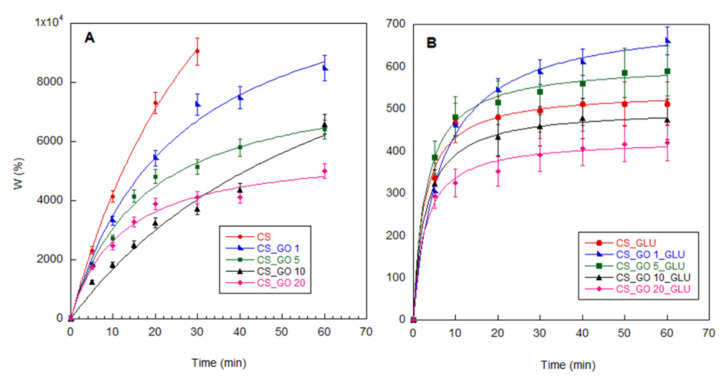
Water-uptake kinetics of non-crosslinked (**A**) and crosslinked (**B**) CS and CS_GOX composites.

Composite materials must show specific behaviour in water, since a good stability is required for the application. Therefore, the water uptake of pristine CS and CS_GO membranes were determined in comparison with the crosslinked membranes (Figure 5). The kinetics of water uptake were collected by measuring, at different times, the increase in weight of the wet samples after their immersion in water. The non-crosslinked membranes showed a high water uptake, since the swelling percentage reached a maximum value of 9000% for pristine CS and 8000% for the CS_GO1 sample. Values from 4000 to 6000% were instead obtained by increasing the GO content. Therefore, the swelling behaviour of the CS-based membranes seemed to be controlled by the GO loading amount. In fact, the decrease in water uptake observed with the filler increase might have been due to the enhancement of CS_GO interactions, resulting in both a reduction in free amino and hydroxyl groups of CS and a limitation in mobility of the polymer chains [40]. Moreover, the presence of graphitic regions in the GO structure made the composites more hydrophobic. Despite this, no dimensional stability was obtained for the membranes, since all non-crosslinked samples dissolved after 1 h of immersion in water (a time of 30 min was sufficient for the pristine CS). On the contrary, no phenomena of dissolution occurred when the composite membranes were crosslinked with glutaraldehyde. Moreover, a further reduction in water uptake was observed for the crosslinked samples due to the lower availability of the amino groups engaged in bonding with glutaraldehyde—the groups mainly responsible for interactions of CS with water molecules. The swelling kinetics of the crosslinked samples showed a characteristic behaviour, with a quick increase of water uptake in the first 10 min of analysis, after which a constant swelling value was reached. The influence of the filler was evident at high GO content. Indeed, it was possible to note a more pronounced reduction in water uptake only for the CS_GO10_GLU and CS_GO20_GLU samples. Oddly, the swelling of the CS_GLU membrane was lower than those of the CS_GO1_GLU and CS_GO5_GLU samples. This might have been due to the formation of a more compact structure in the crosslinked CS that limited water permeability. Therefore, to ensure a better dimensional stability of the membranes in aqueous medium, the presence of both high GO content and a crosslinking reaction are necessary.

The increase in CS hydrophobicity due to the GO presence was also confirmed by contact-angle measurements (Table 1). Indeed, all of the CS_GOX samples showed contact-angle values higher than CS. According to the TGA and water-uptake data, the contact angle values also evidenced a further reduction in hydrophilicity of the composite membranes after the crosslinking reaction. Specifically, a more significant increase in hydrophobicity was reached by the CS_GO10_GLU and CS_GO20_GLU samples.

### 2.6. Pesticide Extraction

SPE is conventionally performed by packing a porous sorbent in a cartridge. Drawbacks related to occlusion problems (for instance, when “dirty” real samples are processed through a cartridge) can be overcome, for example, by employing dispersive SPE, magnetic SPE, or disk SPE. Carbon nanomaterials have excellent properties that make them ideal candidates as sorbents for solid-phase extraction (SPE). Nevertheless, their large surface-specific area, which is one of their key characteristics, can dramatically decrease due to bundling phenomena. A nanomaterial-based membrane can be used as a disk, either packed in a cartridge operated in flow-through mode, or as a rotating device under the action of magnetic stirring. Compared with SPE in a cartridge, the main advantages that arise in the membrane/carbon nanomaterial combination are the excellent results in terms of transport rates and adsorption capability.

To verify the applicability of the CS_GOX membranes as materials for SPE, selected samples were tested in a preliminary experiment of pesticide extraction. Despite the low mechanical performance, the CS_GLU and CS_GOX_GLU samples were employed to check the effect of the GO content on their sorption capacity towards pesticides, since such membranes were extremely stable in water media. The experiment was carried by using 20 pesticides belonging to several classes.

**Figure 6:**
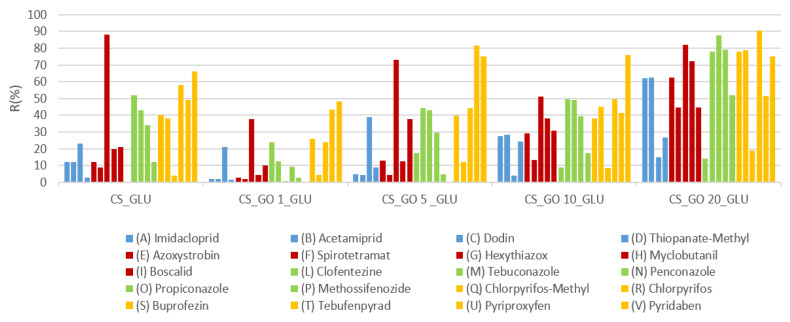
Recovery capacity of the CS_GLU and CS_GOX_GLU composite membranes. Pesticides are reported in ascending order of log K_ow_.

The recovery capacity, expressed in percentage (R%), was obtained for each pesticide and is reported in Figure 6. By observing the R% data, it was possible to verify that the yields depended on the hydrophobicity of the membranes. Generally, pollutants with log K_ow_ between 0.8–2 (pesticides A–D; blue-coloured) showed low R% when CS or CS_GO with low GO content were used (1% and 5%). This could be related to the more hydrophilic nature of those composite membranes (see contact-angle measurements), which allowed a strong interaction with the pollutants, but compromised the extraction phase. Notably, the CS_GLU sample displayed R% values slightly higher than CS_GO1_GLU and CS_GO5_GLU. Most likely, the low permeability of the CS_GLU membrane (see Figure 5) allowed the pesticides’ adsorption, mainly on surface layers, making the extraction easier but still relatively low. Interestingly, when the GO content was high, as for the CS_GO10_GLU and CS_GO20_GLU composites, an enhanced recovery of those pollutants was obtained. To explain this behaviour, a synergic effect between the CS and GO was hypothesized. In fact, thanks to the simultaneous presence of hydrophilic groups (NH_2_ and OH groups of CS, as well as OH and COOH groups of GO) and hydrophobic zones, a suitable interaction between the composite membranes and pesticides with low log K_ow_ was possible. Therefore, by employing the composite membranes with a high GO amount, it was possible to modulate the CS adsorption properties to favour the interactions with hydrophobic pollutants and reduce the interactions with hydrophilic pollutants. A similar behaviour was verified for pesticides with a log K_ow_ value between 2 and 3 (pesticides E,G,H,I; red-coloured) with the exception of Hexythiazox (G), for which a high R% was obtained with all systems. Quite high R% values were registered when testing the pesticides with a log K_ow_ between 3 and 6 (green and yellow colours) for all composites. Interestingly, the increase in the matrix hydrophobicity did not compromise the extraction of more hydrophobic pollutants, which, on the contrary, increased with the increase in GO content. In fact, the best results were achieved by using the CS_GO20_GLU sample as sorbent material. This behaviour suggested how the formation of hydrogen bonds and hydrophobic interactions (π-stacking) could be modulated by varying the GO content into the CS-based membranes, with the advantage of a better pollutant extraction.

So far, as reported in the review by da Silva Alves et al. [22], many studies have been carried out on CS/GO systems for their use as adsorbent materials in environmental applications. To compare the performances of our membranes, particularly of the CS_GO_GLU system, with those of other systems investigated in the literature is very difficult. Indeed, both physical properties of CS (molecular weight, polydispersive index, deacetylation degree, sample batch, and its concentration in solution) and conditions under which the absorption experiments are conducted can be greatly different. In addition, a fundamental parameter that influences the transport phenomena in adsorption processes is the morphology of the used sorbent material. Indeed, a 3D sorbent system, having a porosity and surface area greater than a membrane, could lead to high recovery capabilities for pollutants.

However, in our opinion, this study was very worthwhile in finding a correlation between the amount of GO and the recovery capabilities of the developed systems to be used in the design of nanomaterial-based SPE devices.

## 3. Materials and Methods

### 3.1. Preparation of Chitosan and Chitosan Graphene Oxide Membranes 

Polymer-based membranes are high-performance materials used in various separation processes. Their features, such as morphology, porosity, presence or absence of charges, and composite form, which strongly affect transport phenomena, can be controlled by selecting an appropriate preparation method. Among different techniques that can be used for membrane preparation [45,46], in this work the solution-casting method was employed for preparation of CS-based membranes containing GO in different amounts. In particular, chitosan (CS) with a medium molecular weight (200–800 cP and 75–85% deacetylation degree, purchased from Sigma Aldrich, Darmstadt, Germany, animal origin, CAS number 9012-76-4) was dissolved in 50 mL of 1% *v*/*v* acid acetic solution to obtain a 2% *w*/*v* concentration. The solution was stirred for 24 h. After complete polymer dissolution, the solution was dialyzed into a tube (flat width 43 mm, cut off 14.000) for 24 h to remove excess acetic acid. Since a volume increase was observed during the dialysis process, the polymeric solution was heated to 50 °C to restore the original concentration. For CS membrane preparation, the polymer solution was poured into a petri dish (d = 7 cm) and left at room temperature to remove water. To prepare the CS_GO membranes, GO (powder, density 1.8 g/cm^3^, 15–20 sheets, oxidation 4–10%, Sigma Aldrich, Darmstadt, Germany) at different amounts with respect to CS (1, 5, 10, and 20% *w*/*w*) was suspended in 10 mL of water. Then, the suspension was sonicated for 4 h, mixed with the previously prepared CS solution for 1 h, and poured into a Petri dish for composite membrane preparation. The samples were named CS and CS_GOX, respectively, for pristine chitosan and composite membranes, where X, in the case of composite membranes, referred to the amount of GO dispersed into the polymeric matrix. The surface-crosslinking reaction, carried out to increase the dimensional stability of water in the membranes, was performed by covering the samples with a 0.1% (*w*/*v*) glutaraldehyde solution at room temperature for 30 min. Then, the membranes were washed with water and dried at room temperature. The crosslinked samples were named CS_GLU and CS_GOX_GLU.

### 3.2. Scanning Electron Spectroscopy

Surface and bulk morphologies of all crosslinked or non-crosslinked membranes were investigated by field emission scanning electron microscopy (FESEM, AURIGA Carl Zeiss AG, Oberkochen, Germany). For analysis, the membranes were fractured, gold sputtered, and observed.

### 3.3. Fourier Transform Infrared Spectroscopy

GO interactions with CS and the crosslinking reaction were evaluated by Fourier transform infrared spectroscopy (FTIR). Spectra were acquired in attenuated total reflection (ATR) by a Nicolet 6700 (Thermo Fisher Scientific, Waltham, MA, USA) equipped with a Golden Gate single reflection diamond ATR accessory at a resolution of 4 cm^−1^ and coadding 200 scans. OMNIC^TM^ software for spectra analysis and peak area determination was used.

### 3.4. Thermogravimetric Analysis

The thermogravimetric analysis (TGA) was carried out employing a Mettler TG 50 thermobalance (Mettler Toledo, Columbus, OH, USA). The sample analysis was performed under N_2_ flow, in the temperature range of 25–500 °C, by using a heating rate of 10 °C/min. Two or three temperature scans were carried out on all of the samples. In addition, in the case of crosslinked composites, several pieces of the same polymer membranes were subjected to TGA measurements.

### 3.5. Mechanical Analysis

The mechanical properties of the CS, CS_GOX, and CS_GOX_GLU membranes were studied by tensile tests using an ISTRON 4502 instrument (Instron Inc., Norwood, MA, USA). The samples, which had a rectangular shape (400 × 50 × 0.2 mm, length × width × thickness), were placed between the two Instron flat plates and tested by using a 2 KN load cell at a constant deformation rate of 10 mm/min. The elastic modulus, tensile strength and elongation at break were determined for all of the samples.

### 3.6. Water-Uptake Kinetics

To determine water uptake, weighted membranes (W0) were immersed in distilled water at room temperature. At different times, the samples were taken, lightly dabbed on filter paper, and weighted (Wt) again. The water uptake (W%) was defined as follows:

(1) $$\;W\left(\%\right)=\frac{\mathrm{Wt}-W0}{W0}\;\times 100$$

Five different samples were tested, and the results are reported as average value ± standard deviation.

### 3.7. Static Contact-Angle Measurement

Static contact-angle measurements were carried out by using the drop method. Briefly, for the CS and CS_GOX membranes, a water drop (Milli-Q water) was laid on the membrane surface and a picture was taken. As for the crosslinked membranes, before droplet deposition, the CS_GOX_GLU samples were put under a weight for two days to obtain flat surfaces. SigmaPlot (Systat Software Inc., San Jose, CA, USA) was used as the image-elaboration program. Drop base length (D) and drop height (h) were measured, and the contact angle was determined as follows:

(2) $$\theta =2\;\tan^{-1}\frac{2\;h}{D}$$

Each reported contact angle was the mean value of five measurements collected in different locations on the specimen surface.

### 3.8. Procedure for SPE of Pesticides

#### 3.8.1. Pesticide Mixture Preparation

Analytical standards, all with a purity greater than 98%, were purchased from Aldrich–Fluka–Sigma S.r.l. (Milan, Italy) (Table 2). Individual stock solutions were prepared by dissolving weighed amounts of the analyte standards (pesticides) in appropriate solvents: acetonitrile for clofentezine, toluene for pyraclostrobin, and methanol for other analyte standards. The concentration of all the stock solutions was 1 mg/mL, except that of piraclostrobin, which was 0.5 mg/mL. Composite standard solutions were obtained by diluting the individual ones with methanol at concentrations depending on the purpose. All of the standards and solutions were stored at 4 °C.

#### 3.8.2. Pesticide Extraction

**Figure 7:**
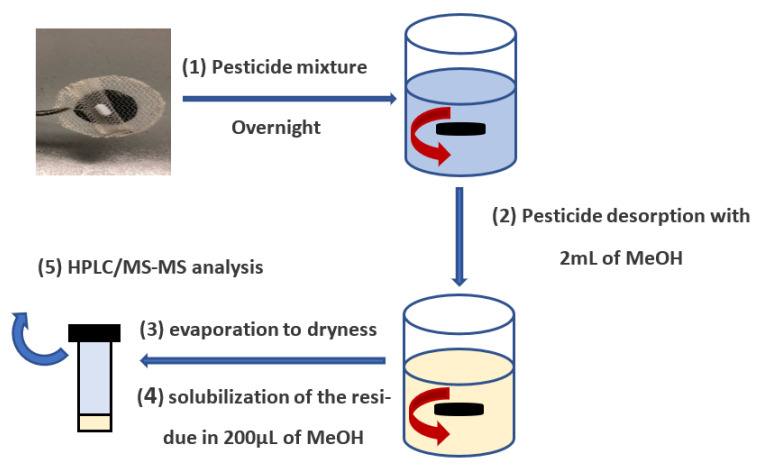
Scheme of the pesticide-extraction procedure.

A specific device, a rotating-disk SPE developed by Tomai et al. [47], containing about 40 mg of membrane, was immersed in the pesticide solution (50 mL, 5 µg/L). The system was left under magnetic stirring overnight for 16 h at 100 rpm to favor analyte adsorption on the membrane. Afterwards, to remove the excess of water, the device was dabbed with a filter paper and the analytes were extracted with two 2 mL fractions of methanol under magnetic stirring (100 rpm). The contact time for each fraction was 30 min. The volume of each single fraction was enough to completely cover the composite membrane. The two organic fractions were collected into a 15 mL falcon tube and evaporated to dryness at 40 °C under nitrogen flow. Finally, the residue was dissolved in 200 μL of methanol, and 2 μL was injected into the HPLC-MS/MS system. The pesticide extraction procedure is reported in Figure 7.

#### 3.8.3. Pesticide Analysis

The extracts were analysed by means of a HPLC series 200 binary pump equipped with an autosampler (Perkin Elmer, Norwalk, CT, USA). The column was an X-Terra C18 (2.1 × 150 mm; 3.5 µm), protected by a guard column (Waters, Milford, MA, USA). The mobile phase was composed of water (phase A) and acetonitrile (phase B), both being 5 mM in formic acid. The analyte separation was carried out by applying a flow rate of 0.200 mL min^−1^ and eluting in linear gradient: phase B was increased from 35% to 100% in 16 min, and then held at 100% for 4 min, for a total run time of 20 min. The autosampler needle device was washed with the phase B after each injection. The detection was performed with an API 4000 Qtrap mass spectrometer (AB SCIEX, Foster City, CA, USA) equipped with an electrospray source using the following settings: capillary voltage +5000 V, air nebulizer gas 2 L min^−1^, air-drying gas at 350 °C and 20 L min^−1^, nitrogen curtain gas 5 L min^−1^, nitrogen collision gas 4 mTorr. The full width at half maximum (FWHM) was set at *m*/*z* 0.7 ± 0.1 in each mass-resolving quadrupole to operate with a unit resolution. Chromatograms were acquired using the multiple-reaction monitoring (MRM) scan mode. Recovery capacity (R%) was expressed in percentages and determined considering the peak area of the extracted pesticide with respect to the peak area of the initial amount.

## 4. Conclusions

With the aim to produce a versatile sorbent material for SPE, the hydrophilicity of chitosan-based membranes was modulated by the introduction of different amounts of graphene oxide (from 1% to 20%, *w*/*w*), used as a filler. The dimensional stability in aqueous medium of the composite membranes was increased by surface crosslinking reaction with glutaraldehyde. It was observed that GO miscibility with the polysaccharide strongly depended on the filler content. SEM analysis revealed the formation of more homogeneous composite membranes at low GO percentages, while filler aggregates were detected with an increase in the GO content. Interactions between the CS and GO components were demonstrated by FTIR measurements, as well as the formation of a Schiff base after the crosslinking reaction. The different filler dispersion also influenced the thermal properties of CS. The CS_GO1 sample showed a degradation temperature higher than that of the pristine CS, whereas a further increase in the GO content modified the polymer chain packing, causing a decrease in T_d_ values. The same trend, with T_d_ values shifted at higher temperatures due the formation of chemical bonds, was shown by the CS_GO_GLU samples after the crosslinking reaction. The GO introduction also enhanced mechanical properties of the CS-based membranes, while the subsequent crosslinking reaction increased their brittleness. Despite GO reducing hydrophilicity of the polysaccharide, as demonstrated by contact-angle and swelling measurements, only the crosslinking reaction made the CS_GOX_GLU samples stable in aqueous medium. It was evidenced that the pesticide-extraction capacity of the developed membranes was effectively influenced by the amount of graphene oxide. Indeed, the R% of different pesticides benefited from the increase of the membrane hydrophobicity caused by the filler content. Specifically, the presence of GO reduced the affinity of the composite membranes for the most hydrophilic pollutants, and considerably increased the R% for the hydrophobic pollutants, particularly in the case of the highest GO content. Therefore, the CS_GO20_GLU sample, which showed the best performance, was able to reach the highest recovery capacity, asserting itself as an innovative, lower-cost, and effective material for the preconcentration of a wide range of pollutants.

## Figures and Tables

**Table 1:** Physical properties of the CS_GO and CS_GO_GLU composites.

Samples	GO/CS (*w*/*w* %)	A_1640_/A_1540_ *	Contact Angle (ϑ°)	T_d_ (°C) **	Weight Loss (%)		Mechanical Properties ***		
					25°–170° (°C)	170°–500° (°C)	E (GPa)	TS (MPa)	EB
CS	0	0.31	84 ± 3	276	16	56	1.4	60	0.2
CS_GO1	1	0.31	85 ± 2	280	15	50	1.4 ± 0.5	53 ± 8	0.20 ± 0.04
CS_GO5	5	0.34	86 ± 2	277	17	51	1.50 ± 0.03	52 ± 7	0.12 ± 0.06
CS_GO10	10	0.34	91 ± 1	276	15	51	1.8 ± 0.4	47 ± 5	0.10 ± 0.02
CS_GO20	20	0.36	92 ± 3	272	10	45	2.7 ± 0.2	63 ± 7	0.09 ± 0.02
CS_GLU	0	0.49	94 ± 3	268	20	53	-	-	-
CS_GO1_GLU	1	0.49	93 ± 4	286	13	51	0.18 ± 0.04	14 ± 4	0.04 ± 0.01
CS_GO5_GLU	5	0.46	92 ± 1	281	11	52	0.48 ± 0.06	24 ± 4	0.04 ± 0.01
CS_GO10_GLU	10	0.43	95 ± 2	281	8	50	0.51 ± 0.03	32 ± 2	0.05 ± 0.01
CS_GO20_GLU	20	0.38	98 ± 2	279	9	46	0.55 ± 0.04	36 ± 6	0.17 ± 0.05

* A_1640_/A_1540_ = area ratios of peaks at 1640 cm^−1^ and 1550 cm^−1^; ** T_d_ = degradation temperature. *** Mechanical properties: E = Young’s modulus; TS = tensile strength; EB = elongation at break.

**Table 2:** Analytical standards (pesticides) used for the extraction with CS_GO composite membranes.

Pesticide	log K_ow_	Pesticide	log K_ow_ *
(A) Acetamiprid	0.8	(M) Tebuconazole	3.70
(B) Imidacloprid	1.1	(N) Penconazole	3.72
(C) Dodin	1.25	(O) Propiconazole	3.72
(D) Thiopanate-Methyl	1.40	(P) Methossifenozide	3.72
(E) Azoxystrobin	2.50	(Q) Chlorpyrifos-Methyl	4.00
(F) Spirotetramat	2.51	(R) Chlorpyrifos	4.12
(G) Hexythiazox	2.67	(S) Buprofezin	4.93
(H) Myclobutanil	2.89	(T) Tebufenpyrad	4.93
(I) Boscalid	2.96	(U) Pyriproxyfen	5.37
(L) Clofentezine	3.10	(V) Pyridaben	6.37

* K_ow_ = octanol/water partition coefficient expressed as the concentration of a species into octanol phase versus the concentration of a species into water phase.

## Data Availability

Data are contained within the article.
